# Novel insight into the relationship between organic substrate composition and volatile fatty acids distribution in acidogenic co-fermentation

**DOI:** 10.1186/s13068-017-0821-1

**Published:** 2017-05-26

**Authors:** Huijun Ma, He Liu, Lihui Zhang, Meng Yang, Bo Fu, Hongbo Liu

**Affiliations:** 1 School of Environmental and Civil Engineering, Jiangnan University, Wuxi, 214122 China; 2 Jiangsu Key Laboratory of Anaerobic Biotechnology, Wuxi, 214122 China

**Keywords:** Waste activated sludge, Substrate composition, VFA distribution, Microbial community, Acidogenic co-fermentation, Alkaline pH

## Abstract

**Background:**

Co-fermentation is an attractive technology for improving volatile fatty acids (VFAs) production by treatment of solid organic wastes. However, it remains unclear how the composition of different organic matters in solid waste influences the VFAs distribution, microbial community structure, and metabolic pathway during acidogenic co-fermentation. In this study, different organic wastes were added into waste activated sludge (WAS) as co-fermentation substrates to explore the impact of organic matter composition on VFAs pattern and the microbiological mechanism .

**Results:**

Acetate was the most dominant VFA produced in all fermentation groups, making up 41.3–57.6% of the total VFAs produced during acidogenic co-fermentation under alkaline condition. With the increased addition of potato peel waste, the concentrations of propionate and valerate decreased dramatically, while ethanol and butyrate concentrations increased. The addition of food waste caused gradual decreases of valerate and propionate, but ethanol increased and butyrate was relatively stable. Some inconsistency was observed between hydrolysis efficiency and acidification efficiency. Our results revealed that starch was mainly responsible for butyrate and ethanol formation, while lipids and protein favored the synthesis of valerate and propionate. Microbial community analysis by high-throughput sequencing showed that Firmicutes had the highest relative abundance at phylum level in all fermentation groups. With 75% potato peel waste or 75% food waste addition to WAS, Bacilli (72.2%) and Clostridia (56.2%) were the dominant respective classes. In fermentation using only potato peel waste, the Bacilli content was 64.1%, while the Clostridia content was 53.6% in the food-only waste fermentation.

**Conclusions:**

Acetate was always the dominant product in acidogenic co-fermentation, regardless of the substrate composition. The addition of carbon-rich substrates significantly enhanced butyrate and ethanol accumulation, while protein-rich substrate substantially benefited propionate and valerate generation. Potato peel waste substantially favored the enrichment of Bacilli, while food waste dramatically increased Clostridia content in the sludge.

**Electronic supplementary material:**

The online version of this article (doi:10.1186/s13068-017-0821-1) contains supplementary material, which is available to authorized users.

## Background

With increasing energy demands and a shortfall in renewable resources, many studies of energy utilization are now aimed toward resource recovery. Common targets include biogas and volatile fatty acids (VFAs) from agricultural residues, food waste, and sewage sludge containing high levels of organic matter [1, 2]. The large amounts of proteins, polysaccharides, and other types of organic matter in waste activated sludge (WAS) can be efficiently hydrolyzed to water-soluble organic substances by fermentative microorganisms, thus achieving resource recovery [3, 4]. VFAs, the main intermediates during anaerobic fermentation, have been recognized as a valuable carbon resource in wastewater, and can be used as platform molecules to produce biopolymers and medium chain fatty acids with high added value [5–7]. In addition to the improvement of VFA yields during anaerobic fermentation of solid wastes [3, 8], it is also important to control the composition of VFAs and to achieve selective VFA production to benefit downstream processing and applications.

An increasing number of studies are now using co-fermentation to improve the VFAs product yield from anaerobic sludge fermentation. However, there is little information on how the complex organic matter content influences the VFAs distribution and the microbial mechanism that proceeds during co-fermentation. For example, VFAs yields were 1.75-, 10.7-, and 2.6-fold higher than WAS fermentation alone, when food waste, perennial ryegrass, or henna plant was used as co-fermentation substrate, respectively, with a carbon-to-nitrogen ratio (C/N) of about 22/1 [9–11]. Interestingly, reported patterns of VFAs in previous co-fermentation studies were different, even when the substrate types and addition ratios were similar. Reported VFAs yields were twofold [12] and 10.6-fold [13] higher than for sludge fermentation alone when the mixing ratio of food waste to sludge was 50% during co-fermentation. Some studies have attributed the increased VFAs yields to the involvement of organic matter and the microbial community [14, 15]. For example, when adding different types of substrates, some major phyla, such as Firmicutes, Chloroflexi, and Proteobacteria, were significantly changed [16].

These previous studies hinted that the composition of the organic matter in solid wastes significantly influenced the composition of the fermented VFAs. However, research has been lacking on the detailed relationship between the organic matter and the distribution of VFAs, and on how the organic composition influences the microbial community or metabolic pathway. This knowledge gap has hindered the industrial application of selective VFA production process.

This study investigated the effects of different organic matters on the VFAs metabolic pathway and the microbial community shift during the co-fermentation of sludge and carbon-rich organic wastes. Potato peel waste and food waste, the two common carbon-rich substrates used in China, were added into WAS in different proportions for anaerobic fermentation. The release and consumption of different organic components were studied along with the VFAs product yields and composition changes. Shifts in microbial community structure and functional community evolution under the different experimental conditions were studied by high-throughput sequencing analysis.

## Methods

### Characteristics of WAS, potato peel waste, and food waste

WAS used in fermentation was dewatered sludge obtained from a municipal wastewater treatment plant in Wuxi, China (Shuofang wastewater treatment plant, Wuxi, Jiangsu). Potato peel waste was obtained from a vegetable market in Wuxi, and food waste was collected from the canteen of Jiangnan University. The food waste mainly consisted of fat, rice, meat, and vegetables. Potato peel waste and food waste were fully crushed by a food crusher after bones and other hard objects were removed. WAS, crushed potato peel waste, and crushed food waste were stored at 4 °C for subsequent fermentation experiments. Composition and properties of the wastes are shown in Table 1. The lipid content in potato peel waste was below the detection limit, and its concentration was neglected in the following calculations of organic consumption.

**Table 1:** Characteristics of WAS, potato peel waste, and food waste

Parameters	WAS	Potato peel waste	Food waste
Solid content (%)	14.54 ± 0.10	20.59 ± 1.42	35.12 ± 1.03
VS/TS (%)	45.85 ± 0.29	94.62 ± 0.61	93.68 ± 0.47
pH	6.60 ± 0.08	6.01 ± 0.12	5.08 ± 0.16
TCOD (mg/g TS)	849.29 ± 19.45	1292.45 ± 28.94	1497.32 ± 39.23
SCOD (mg/g TS)	262.78 ± 6.41	543.20 ± 2.02	618.28 ± 7.13
TN (mg/g TS)	25.90 ± 1.24	4.53 ± 0.07	10.53 ± 1.06
Protein (mg/g VS)	323.96 ± 5.86	69.81 ± 5.60	123.03 ± 2.56
Carbohydrate (mg/g VS)	305.34 ± 12.32	792.23 ± 10.34	284.65 ± 5.54
Starch (mg/g VS)	183.74 ± 3.12	673.44 ± 4.36	214.50 ± 1.34
Lipid (mg/g VS)	93.73 ± 0.45	*	564.23 ± 1.54
VFAs (mg/g VS)	18.27 ± 1.20	20.82 ± 1.35	27.94 ± 1.84

* Not detected

Seeding sludge was obtained from the Shuofang wastewater treatment plant, and an upflow anaerobic sludge blanket (UASB) reactor (effective volume ~5.0 L) was used for acidogenic bacteria acclimation. The WAS was heated at 105 °C for 2 h to kill non-spore-forming methanogens. Before being used as sludge inoculum, the heat-treated sludge was added to the UASB reactor for re-activation. Glucose was continuously pumped into the UASB to enrich the acidogenic bacteria during the operation, and the temperature was maintained at 37 ± 2 °C. Seeding sludge was obtained when the effluent pH fell below 4.0. The cultivation period was more than 3 weeks [17]. Characteristics of the seeding sludge were as follows: total solids (TS) 54.90 ± 0.72 g/L, volatile solids (VS)-to-TS ratio (VS/TS) 85.72 ± 1.23%, protein 653.20 ± 10.01 mg/g TS, carbohydrate 208.41 ± 80.21 mg/g TS, and VFAs 3.45 ± 0.23 g/L. The cultivated seeding sludge was washed three times with deionized water before use in the fermentation reactors.

### Experimental set-up

Prior to fermentation, WAS was pretreated by heat and alkali to accelerate the hydrolysis of organic matter. WAS was heated at 105 °C for 3 h, and pH was adjusted to 12 with the addition of 5 M NaOH. A pH of 10 was maintained during fermentation by daily addition of 2 M HCl or 2 M NaOH to inhibit the activity of methanogenic bacteria. To ensure that enough acidogenic microorganisms were in the fermentation reactor, an amount of seeding sludge (~15% of VS) was added to control the food–microorganism ratio (F/M) to about 5.67 before the tests started. Nine 500-mL serum bottles were placed in an air-bath shaker (35 ± 1 °C, 120 rpm) for anaerobic fermentation. According to the substrate contents in each bottle, they were labeled as OS (only pretreated sludge as substrate), OP (only potato peel waste as substrate), OF (only food waste as substrate), SP1 (VS ratio of pretreated sludge and potato peel waste = 3:1), SP2 (VS ratio of pretreated sludge and potato peel waste = 1:1), SP3 (VS ratio of pretreated sludge and potato peel waste = 1:3), SF1 (VS ratio of pretreated sludge and food waste = 3:1), SF2 (VS ratio of pretreated sludge and food waste = 1:1), SF3 (VS ratio of pretreated sludge and food waste = 1:3). The total VS concentration of organic substrates before fermentation was set to about 30 g/L and the fermentation was conducted in batch operation for 12 days. The experimental set-up, as well as the initial organic matter concentrations, are shown in Table 2. Before acidogenic fermentation, 400 mL of substrate and seeding sludge was added into each reactor and 50 mM 2-bromoethanesulfonic acid sodium salt was added to inhibit methanogens. Each reactor was sealed with a rubber stopper and nitrogen was purged for 15 min to displace oxygen in the headspace. The nitrogen purge was conducted at the beginning of each fermentation and during each sample collection.

**Table 2:** Sample set-up and concentrations of organic materials before fermentation

Fermentation groups	OS	OP	OF	SP1	SP2	SP3	SF1	SF2	SF3
Total VS (g/L)	30.25 ± 0.54	32.03 ± 1.02	30.33 ± 0.72	28.01 ± 0.20	29.63 ± 0.57	30.43 ± 0.89	31.46 ± 1.06	29.45 ± 0.93	30.48 ± 0.23
C/N	10.23 ± 0.53	44.43 ± 0.92	80.44 ± 1.23	17.37 ± 0.45	24.75 ± 0.67	45.02 ± 0.66	14.34 ± 0.26	21.21 ± 0.43	30.43 ± 1.02
Total protein concentration (mg/L)	9799.79 ± 80.34	22,36.01 ± 31.57	3731.50 ± 33.24	7494.43 ± 60.83	5833.70 ± 60.23	3857.76 ± 49.22	8011.47 ± 75.56	6081.93 ± 54.88	4581.04 ± 39.27
Total starch concentration (mg/L)	8666.32 ± 129.34	21,570.28 ± 306.44	6505.78 ± 89.35	9734.20 ± 150.73	13,221.36 ± 219.33	17,549.05 ± 283.76	7314.37 ± 113.55	7389.61 ± 102.34	7006.52 ± 104.31
Total lipid concentration (mg/L)	2835.33 ± 30.43	0.00	17,113.10 ± 291.34	1470.62 ± 20.34	1288.61 ± 18.20	703.05 ± 7.46	6249.22 ± 149.39	9688.46 ± 172.34	13,912.52 ± 281.44

### Analytical methods

All samples collected during fermentation were 10.0 mL in volume, and were analyzed immediately after collection. Determination of TS, VS, soluble chemical oxygen demand (SCOD), total COD (TCOD), and total nitrogen (TN) were conducted according to standard methods [18]. Total lipid and total starch were analyzed according to Chinese standard methods [19]. Protein concentrations were measured using the Lowry-Folin method [20]. The C/N ratio was calculated through the ratio of total organic carbon (TOC) to TN. TOC was detected with a TOC analyzer (LiquiTOC, Elementar Analysensysteme, Langenselbold, Germany). To measure soluble COD, samples were first centrifuged at 10,200×*g* for 10 min, and then filtered with 0.45-μm syringe filters.

VFAs concentrations in the filtrate samples were detected by gas chromatography (GC-2010, Japan) with flame ionization detection (FID). Separation was achieved on a fused-silica capillary column (PEG-20M, 30 m × 0.32 mm × 0.5 mm, China). The initial column temperature was 80 °C, and then it was heated to 210 °C and held for 2 min. The running temperature was maintained at 80 °C during detection. The injection port and detector temperatures were 250 °C. Before GC measurement, 4-methyl-valeric acid (internal standard), 3 M phosphoric acid (acidifier), and the filtrate sample were mixed in a 1:1:1 ratio (v:v:v). This method was the same as used in previous studies [21].

Calculations of the hydrolysis and acidification efficiencies were performed according to the following equations:

1 $${\text{Hydrolysis efficiency }}\left( \% \right) \, = \, \left( {{\text{SCOD}}_{\text{final}} - {\text{SCOD}}_{\text{initial}} } \right)/{\text{total COD}}$$

2 $${\text{Acidification efficiency }}\left( \% \right) \, = {\text{ COD}}_{\text{VFA}} /{\text{total COD}},$$

where SCOD_final_ is the SCOD at the end of the fermentation, mg/L; SCOD_initial_ is the SCOD before fermentation, mg/L; and COD_VFA_ is VFA concentration as COD concentration, mg COD/L. The transformation of VFA to COD was conducted as in previous studies [22].

### DNA extraction

Samples of OS, OP, OF, SP3, and SF3 were preserved at the end of fermentation for microbial analysis. The total DNA of the five samples was extracted with a MoBio PowerSoil DNA Isolation Kit (Mo Bio Laboratories, Carlsbad, CA, USA) according to the manufacturer’s instruction, and as used in a previous study [23]. The quantity and quality of the extracted DNA were checked with a Nanodrop 2000 spectrophotometer (Thermo Scientific, Schaumburg, IL, USA). The two primers used for sequencing on the Illumina Miseq sequencing platform were 515F (5′-GTGCCAGCMGCCGCGG-3′) and 907R (5′-CCGTCAATTCMTTTRAGTTT-3′). The raw sequence data were deposited in the NCBI Sequence Read Archive (SRA) database, and the accession number is SRP091729.

### Canonical correspondence analysis

The relationship between bacteria at genus level and environmental parameters was assessed with canonical correspondence analysis **(**CCA) using CANOCO 4.5 software. CCA is an effective method to combine environmental factors to assess how the environment affects research objects (such as bacteria). The arrows in CCA represent environmental factors, and the four quadrants indicate the positive and negative correlation between the environmental factors and the two axes. The length of the arrow represents the connection of environmental factors and the distribution of the samples. For example, longer arrow length indicates a greater influence of environmental factors on the bacteria.

## Results and discussion

### VFAs production and composition during fermentation

**Fig. 1:**
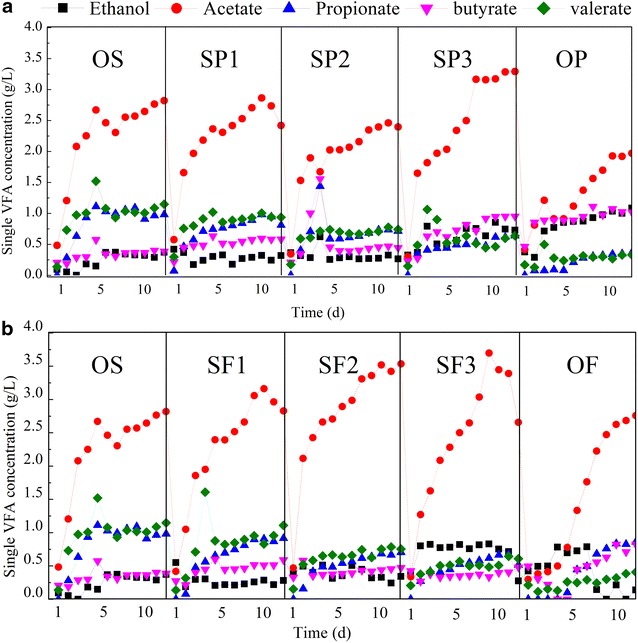
The daily changes of ethanol and four single VFA concentrations during co-fermentation of WAS and potato peel waste (**a**), and WAS and food waste (**b**)

Ethanol and four VFAs (acetate, propionate, butyrate, and valerate) were observed in all fermentation groups (Fig. 1a). In all cases, acetate was produced in large quantities, making up 41.3–47.7% of VFAs content. When fermented with only potato peel waste (OP), the acetate concentration was 1.93 g/L. With an increased potato peel waste addition ratio, the concentration of propionate decreased dramatically from 1.11 to 0.32 g/L and valerate decreased from 1.52 to 0.29 g/L. In contrast, the concentration of ethanol increased from 0.33 to 1.08 g/L and butyrate increased from 0.40 to 1.07 g/L. As a result, the final percentages of acetate, ethanol, butyrate, propionate, and valerate were 46.6, 6.7, 10.9, 16.9, and 18.9%, respectively, in the OS group, and 41.3, 22.6, 21.3, 7.8, 7.0%, respectively, in the OP group.

As shown in Table 1, protein, starch, and lipid were the main organic compounds in WAS, potato peel waste and food waste, respectively. The addition of potato peel waste resulted in increased starch concentration, decreased protein concentration, and enhanced the generation of ethanol and butyrate, while reducing the accumulation of propionate and valerate. Some previous studies reported that when straw [24] or pretreated bagasse [25] was used as co-fermentation substrate, the variation of propionate and butyrate showed a similar trend to that observed in this study. It is likely that the polysaccharides were eventually biodegraded to glucose, similar to the starch substrates used in the present study. It has been verified that glucose fermentation above pH 6.0 produces butyrate and acetate as the main VFA products, while the dominant product is usually ethanol for fermentation under more acidic conditions [26]. This explanation is consistent with the increased accumulation of butyrate and ethanol observed in this study.

As shown in Fig. 1b, when sludge was co-fermented with food waste, acetate was also the dominant VFA produced, with VFAs content ranging between 48.0 and 57.6%, although its concentration decreased significantly to 2.68 g/L in the OF group. As the food waste addition ratio increased, valerate and propionate contents gradually decreased from 1.10 to 0.41 and from 0.86 to 0.49 g/L, respectively. However, ethanol concentration increased from 0.26 to 0.74 g/L and butyrate was relatively stable (0.45–0.51 g/L).

Table 2 shows that the lipid concentration increased from 2835.33 ± 30.43 mg/L in the OS group to 17113.10 ± 291.34 mg/L in the OF group as the ratio of food waste increased, while the valerate and propionate concentrations decreased and ethanol increased. Lipids are mainly biodegraded to fatty acids and glycerol, and glycerol further promotes the generation of H_2_ and ethanol [27]. This might be a reason for the enhanced ethanol generation in the present study. Decreased concentrations of valerate and propionate were observed when potato peel waste and food waste were added, and these changes might be related to low protein concentration. Several reports have suggested that nitrogen-rich substrates enhance propionate and valerate generation during fermentation under alkaline conditions. For example, increased protein consumption would promote propionate generation, and propionate, instead of acetate, was the main product and accounted for 63.4% of total VFAs in the co-fermentation of sludge and food waste [9].

As shown in Fig. 1a and b, acetate was always the major VFA produced, regardless of the composition of the organic matter in the substrate. We speculate that there are two possible reasons for the dominant acetate accumulation. First, the seeding sludge used in this study was domesticated for a long time before the fermentation, and this would have enriched the acidogenic microorganisms in the seeding sludge [28]. Second, alkaline conditions in the fermentation killed or inhibited most of the regular fermentative microorganisms, while the acidogenic bacteria, with most being Gram-positive species and able to generate spores, survived under the harsh alkaline environment [29].

### Organic matter changes during fermentation

Table 3 shows the extent of hydrolysis and acidification, as well as the VFAs yields at the end of each acidogenic fermentation. With the increasing ratio of potato peel waste or food waste, hydrolysis efficiency gradually increased. In the nine fermentation groups, the two highest hydrolysis efficiencies were observed in the OP and OF groups (69.49 ± 1.91 and 54.01 ± 1.34%, respectively). Potato peel waste and food waste were more easily hydrolyzed than WAS, thus improving hydrolysis efficiency.

**Table 3:** Extent of hydrolysis, acidification, and VFAs yields at the end of fermentation

Parameters	OS	SP1	SP2	SP3	OP	SF1	SF2	SF3	OF
Hydrolysis efficiency (%)	49.67 ± 1.67	45.44 ± 1.02	51.17 ± 1.62	56.39 ± 1.53	69.49 ± 1.91	50.35 ± 1.38	48.45 ± 1.82	53.13 ± 1.69	54.01 ± 1.34
Acidification efficiency (%)	14.51 ± 0.53	17.75 ± 0.68	21.25 ± 0.43	26.92 ± 0.46	16.74 ± 0.28	18.23 ± 0.27	20.62 ± 035	23.00 ± 0.64	17.56 ± 0.43
VFAs yield (mg COD/g VS)	132.30 ± 5.43	151.60 ± 6.83	268.40 ± 10.45	343.54 ± 14.63	185.10 ± 7.49	139.67 ± 5.32	217.85 ± 8.47	282.02 ± 6.35	182.52 ± 5.24

For acidification efficiency and VFAs yields, the same trends were observed in all fermentation groups. When potato peel waste was used as substrate for co-fermentation, the highest acidification efficiency (26.92 ± 0.46%) and VFAs yield (343.54 ± 14.63 mg COD/g VS) were observed in the SP3 group. With the addition of food waste, the SF3 group presented the highest acidification efficiency (23.00 ± 0.64%) and VFAs yield (282.02 ± 6.35 mg COD/g VS). The results demonstrated that co-fermentation with sludge and carbon-rich substrates benefited VFAs generation.

Comparing with Table 4, the VFAs yields in this study were similar to those of Jia et al. [10], Rughoonund et al. [25], and Huang et al. [11], which gave the respective figure of 368.71 ± 17.53 g COD/kg TS, 360 mg/g VS, and 7891 ± 411 mg COD/L. However, the studies of Guo et al. [30] and Huang et al. [31] gave higher VFA yields of 486.6 mg COD/g VS and 425.2 mg COD/g VS, respectively, possibly a reflection of the different substrate types. The VFAs yield in the OS group of the present study was only 132.30 ± 5.43 mg COD/g VS, while previous studies using sludge-only fermentation showed VFAs yields of 100–250 mg COD/g VS [36, 37]. The complex composition of the sludge organics may be a reason for the relative low VFAs generation. The low hydrolysis efficiency was the limiting step of acidogenic fermentation, especially in the sludge-only fermentation group (extent of WAS hydrolysis <50%). It is also noted that the fermentation with the highest hydrolysis efficiency (OP) did not show the highest VFAs production, indicating some inconsistency between hydrolysis efficiency and acidification efficiency.

**Table 4:** Comparison of substrates, products, and microbial communities between previous literature studies and this study

Authors	Type of substrates	Target product	Operation mode	Fermentation condition	Highest product yield	Major microorganism	Relative studies (Y to yes, N to no)	
							Substrates composition and microbial community	Microbial community and metabolic pathway
Jia et al. [10]	WAS, perennial ryegrass	VFAs	Batch	35 ± 1 °C, without pH control	368.71 ± 17.53 gCOD/kg TS	*Clostridia*, *Spirochaetes*, and *Bacteroidetes*	Y	N
Rughoonundun et al. [25]	WAS, bagasse	VFAs	Batch	55 °C, pH 7.0	360 mg/g VS	–	N	N
Huang et al. [11]	WAS, henna plant biomass	VFAs	Batch	35 ± 1 °C, initial pH 8.0, without pH control	7891 ± 411 mg COD/L	–	N	N
Guo et al. [30]	WAS, agricultural residues	VFAs	Semi-continuous	pH 10.0 ± 0.5	486.6 mgCOD/g VSS	*Proteobacteria, Firmicutes*, and *Actinobacteria*	Y	N
Huang et al. [31]	WAS, bio-surfactants	VFAs	Batch	pH 9.0, 10.0, and 11.0	425.2 mg COD/g VSS	*Firmicutes, Proteobacteria, Bacteroidetes, Actinobacteria*, and *Chloroflexi*	Y	N
Maspolim et al. [32]	WAS	VFAs	Semi-continuous	35 °C, pH 4, 5, 6, 7, 8, 9, 10, and 11	–	*Tissierella, Petrimonas, Proteiniphilum, Levilinea, Proteiniborus*, and *Sedimentibacter*	Y	N
Sivagurunathan et al. [33]	Galactose	H_2_	Batch and continuous	35 ± 1 °C, pH over 5.5	1.05 mol H_2_/mol galactose	*Clostridium* sp. and *Sporolactobacillus* sp.	Y	Y
Kumar et al. [34]	Microalgae	H_2_	Batch	The seed inoculum included BESA addition (1 g/L), pH 5.5	29.5 mL/g VS_added_	–	N	N
Ho et al. [35]	Microalgal *C. vulgaris FSP*-*E*	Ethanol	Batch	30 °C, pH 5.0-6.0	11.66 g/L	Pure culture of *Z. mobilis ATCC 29191*	N	N
This study	WAS, potato waste, food waste	VFAs	Batch	35 ± 1 °C, pH 10.0	343.54 ± 14.63 mg COD/g VS	*Firmicutes, Chloroflexi*, and *Proteobacteria*	Y	Y

– Not mentioned in literature

**Fig. 2:**
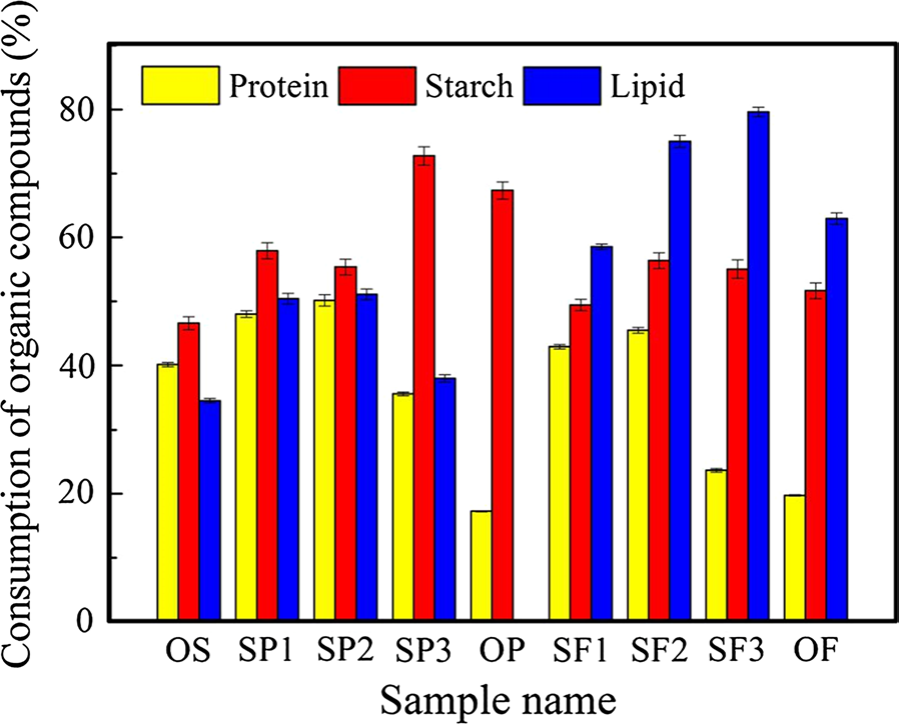
Consumption of different organic materials in the nine fermentation groups

The effects of potato peel waste or food waste on the consumption of different organic matters at the end of fermentation are shown in Fig. 2. When the addition ratio of potato peel waste or food waste was below 50%, protein, starch, and lipid consumption efficiencies were higher than the WAS and carbon-rich substrate fermentations (OS, OP, and OF). For example, the protein consumption of SP1, SP2, SF1, and SF2 groups were 48.02, 50.16, 42.92, and 45.50%, respectively, which were higher than the protein consumption in the WAS and carbon-rich substrate fermentations (40.10% for OS, 17.28% for OP, 19.75% for OF). It is likely that the appropriate addition ratio of carbon-rich substrates could be used to enhance the consumption of organic matter. In addition, a balance of nutrients in fermentation substrates could be used to promote microbial growth and enhance the activity of relevant enzymes. Feng et al. [38] found that addition of carbohydrate substrate promoted the consumption of protein in sludge and promoted VFAs generation because of the activation of enzymes involved in protein hydrolysis.

When the addition of potato peel waste or food waste exceeded 50%, protein consumption decreased dramatically (from 50.16 ± 0.88% in SP2 to 17.28 ± 0.09% in OP and from 45.50 ± 0.45% in SF2 to 19.75 ± 0.10% in OF). However, starch and lipid consumption were enhanced significantly when the addition of potato peel waste or food waste was increased to 75%. This demonstrated that the consumption of carbon-rich substrate predominated over protein consumption in a carbon-rich environment. Moreover, starch and lipid consumption in potato-only and food waste fermentation (OP and OF groups) were less than in SP3 and SF3 groups. Evidently, unbalanced nutrient supply was not beneficial for organic matter consumption.

### Relationship between substrate consumption and VFA generation

**Fig. 3:**
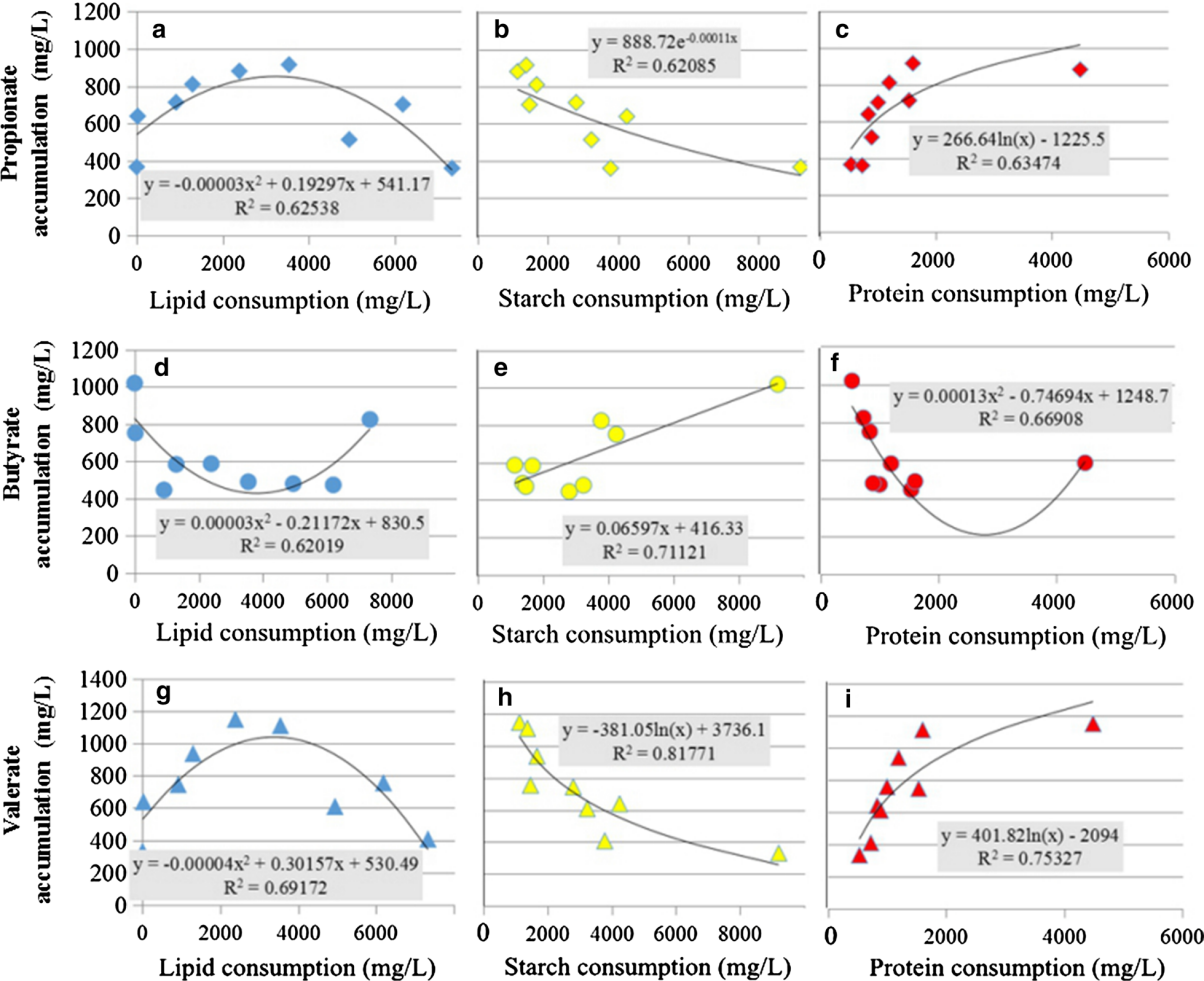
Plots and mathematic relationships between VFAs accumulation and consumption of lipids, starch, and protein during fermentation. **a**–**c** Propionate accumulation; **d**–**f** butyrate accumulation; **g**–**i** valerate accumulation

The relationships between the generation of four different VFAs and the consumption of protein, lipids, and starch at the end of each fermentation were further analyzed. Figure 3a, d, and g shows that the accumulations of propionate, butyrate, and valerate demonstrated quadratic relations with lipid consumption, with the *R* ^2^ values of 0.63, 0.62, and 0.69, respectively. The generation of propionate and valerate was enhanced but butyrate production was decreased when more lipids were consumed. However, the trends in VFAs generation reversed when the lipid concentration exceeded 4000 mg/L. These results indicate that moderate lipid consumption promoted the growth of acidogenic microorganisms, while excess lipid consumption inhibited it. This caused more propionate and valerate accumulation at a lower lipid consumption rate and less propionate and valerate accumulation with increased lipid consumption [39, 40]. Figure 3b, e, and h shows that the generation of propionate and valerate gradually decreased with starch consumption, but butyrate concentration proportionally increased. In relation to protein consumption, propionate and valerate accumulated gradually (Fig. 3c, i) with higher protein consumption, while butyrate concentration declined gradually at low protein consumption, but then increased greatly (Fig. 3f) at higher protein consumption.

According to the above results, starch was mainly responsible for butyrate formation, while lipid and protein consumption favored the synthesis of valerate and propionate. However, different kinds of substrates can be converted to acetate by acidogenic microbes through different metabolic pathways. Therefore, acetate accumulation became dominant regardless of the composition of the substrates in acidogenic fermentation under alkaline conditions. The results in Additional file 1 further implied that pure protein substrate (BSA group) gave a VFA distribution similarity to that of the WAS fermentation group, while the glucose (typical carbohydrate) substrate in acidogenic fermentation gave a VFA distribution similarity to that of the potato-only fermentation group. This demonstrated that fermentation with glucose mainly produced acetate and butyrate while fermentation with bovine serum albumin (BSA) preferentially improved valerate and propionate production, apart from the acetate accumulation.

### Diversity of bacterial community

**Fig. 4:**
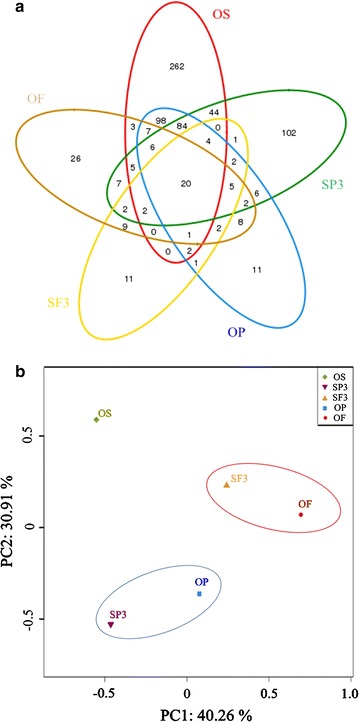
OTUs and the bacteria phylum distribution at the end of fermentation. **a** Venn diagram analysis of the OS, OP, OF, SP3, and SF3 experiment groups; **b** PCA of the OS, OP, OF, SP3, and SF3 experiment groups

As a measure of bacterial diversity, the numbers of shared operational taxonomic units (OTUs) among OS, SP3, OP, SF3, and OF fermentation groups were calculated by Mothur’s Venn diagram analysis (Fig. 4a). Because most bacteria could not survive in an alkaline fermentation environment, a low OTU number of 733 was observed in this study [32]. The total OTUs were 659, 464, 354, 130, and 77 in OS, SP3, OP, OF, and SF3 samples, respectively. Only 20 OTUs (2.7%) were shared by the five samples, indicating that there was an obvious bacterial variety over the five groups. OS, SP3, and OP groups shared 28 OTUs (4.2% of OS, 6.0% of SP3, and 36.4% of OP); OS, SF3, and OF shared 34 OTUs (5.2% of OS, 9.6% of SF3, and 26.2% of OF). The number of OTUs in the OS group was the highest of the five fermentation groups, indicating that the OS sample had the most abundant microbial populations. The Shannon–Weaver index of the OS sample (4.09) was also the highest among all the groups (2.25 for SP3, 1.90 for SF3, 1.98 for OP, and 2.40 for OF). This was because of the relative abundance of protein in the OS sample when compared with SP3, OP, SF3, and OF, which would lead to the survival of more protein-consuming bacteria.

Principal component analysis (PCA) of classified OTUs revealed that the microbial communities in SP3, OP, SF3, and OF fermentation groups were significantly shifted from that in the OS group (Fig. 4b). Relatively similar communities occurred in OP and SP3, and in OF and SF3, but not among the four tests. This finding was further supported by the results of taxonomic analysis. The different classification groups in the five groups clearly revealed the significant impact of organic matter composition on microbial similarity during fermentation.

**Fig. 5:**
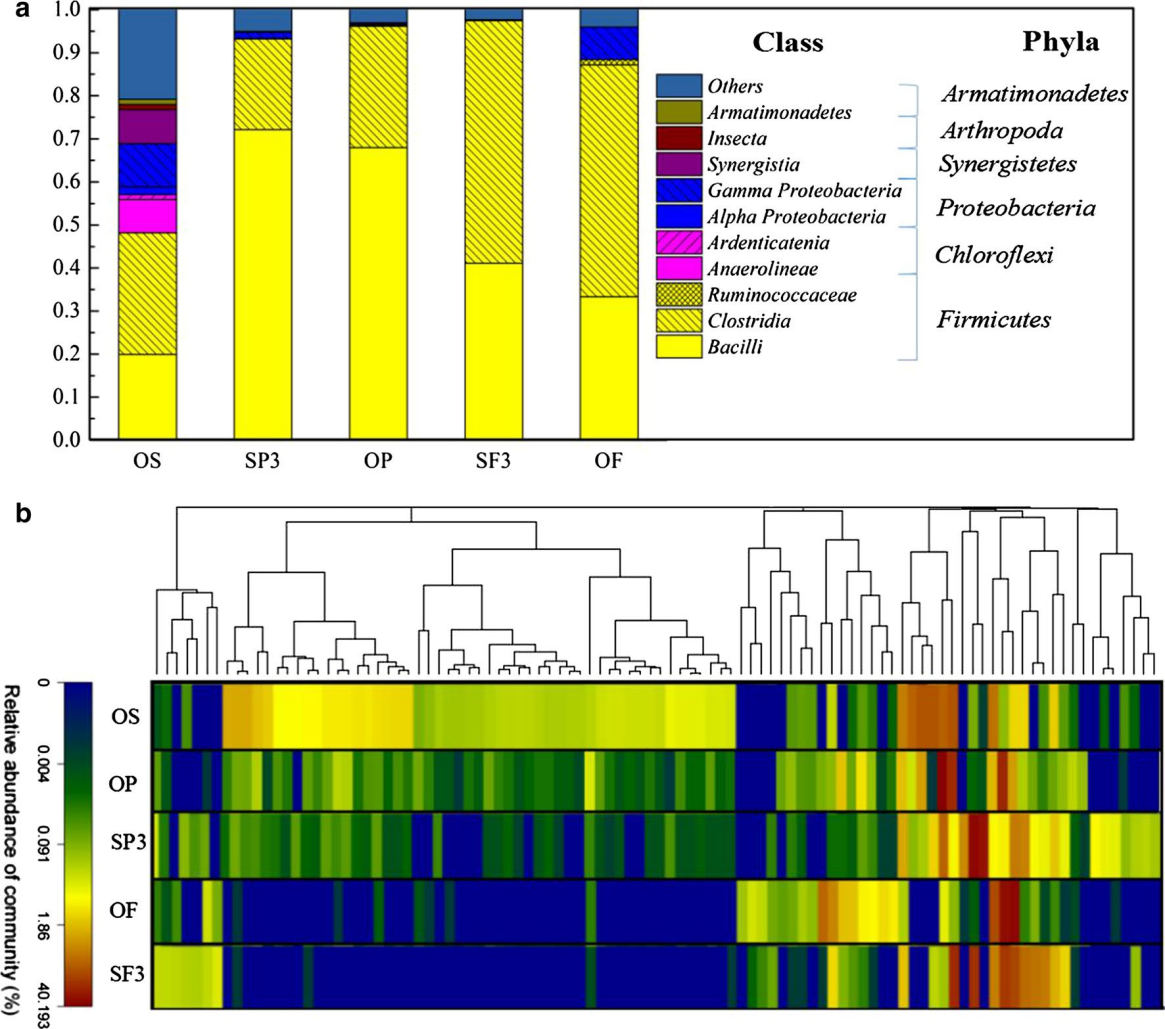
Taxonomic classification of sequences. **a** Bacterial communities of the OS, OP, OF, SP3, and SF3 experiment groups at class level and phylum level. **b** Hierarchical clustering analysis at genus level of bacterial communities of the OS, OP, OF, SP3, and SF3 fermentation groups

The distribution of the bacterial community further explained the differences among OS, OF, OP, SF3, and SP3 groups in detail (Fig. 5a). Five groups showed relatively low diversities with a total of 7 identified phyla observed. The phyla Firmicutes, Chloroflexi, and Proteobacteria, which were recognized as common fermentative phyla [41], were dominant in all five communities with a combined contents of 84.0, 98.9, 99.7, 98.1, and 97.5% in OS, OF, OP, SF3, and SP3, respectively. However, the distribution of the three phyla in the five groups was obviously different. Firmicutes showed the highest relative abundance in the five groups. From Table 4, Firmicutes, Chloroflexi, and Proteobacteria could always be enriched under alkaline conditions, and these bacteria mainly participated in VFAs generation.

At the class level, it was observed that Clostridia (28.2%), Bacilli (11.7%), Gammaproteobacteria (10.0%), Synergistia (7.9%), and Anaerolineae (7.8%) were the five main classes in the OS group, while Bacilli and Clostridia were the two major classes in the SP3, OP, SF3, and OF groups. The abundance of the Bacilli class increased dramatically when potato peel waste was added in the sludge (72.2% in SP3 and 64.1% in OP), while the Clostridia class rose significantly with the addition of food waste (56.2% in SF3 and 53.6% in OF). Some studies have reported that bacteria from Clostridia and Bacilli were able to produce acetate; however, Clostridia bacteria can also produce butyrate [42], and Bacilli bacteria can produce lactic acid [43]. The distribution of special bacterial class in different fermentation groups clearly proved that the composition of organic matter could significantly influence the bacterial community structure, and selectively enrich specific acidogenic bacteria during anaerobic co-fermentation.

To further explore the microbial diversity in the five groups, the results of hierarchical clustering analysis at the genera level are shown in Fig. 5b (genera with relative abundance >1% in each sludge sample are listed in Additional file 2). Relatively similar communities occurred in groups SP3 and SF3, and in OP and OF. Nevertheless, the bacterial community in the OS group was different from the other four groups. This further proves that the composition of organic matter influences and shifts the bacterial community structure at genus level.

### Relationship between substrate composition and bacteria community

Previous studies have explored the relationship between microbial activity and different types of substrates in co-fermentation (Table 4). For example, Guo et al. [30] found that bacteria like Proteobacteria and Firmicutes were enriched under alkaline conditions with added agricultural residue. Moreover, use of perennial ryegrass or fermentation with only WAS gave different microbial communities [10]. Several researchers have also studied different microbial communities and their metabolic pathways based on a single type of substrate. For example, Sivagurunathan et al. [33] reported that two *Clostridium* strains showed different metabolic pathways in different operation modes with galactose as substrate. However, little attention was paid to the relationship between the organic matter composition and the microbial mechanism, or details such as the microbial community and metabolic pathway.

**Fig. 6:**
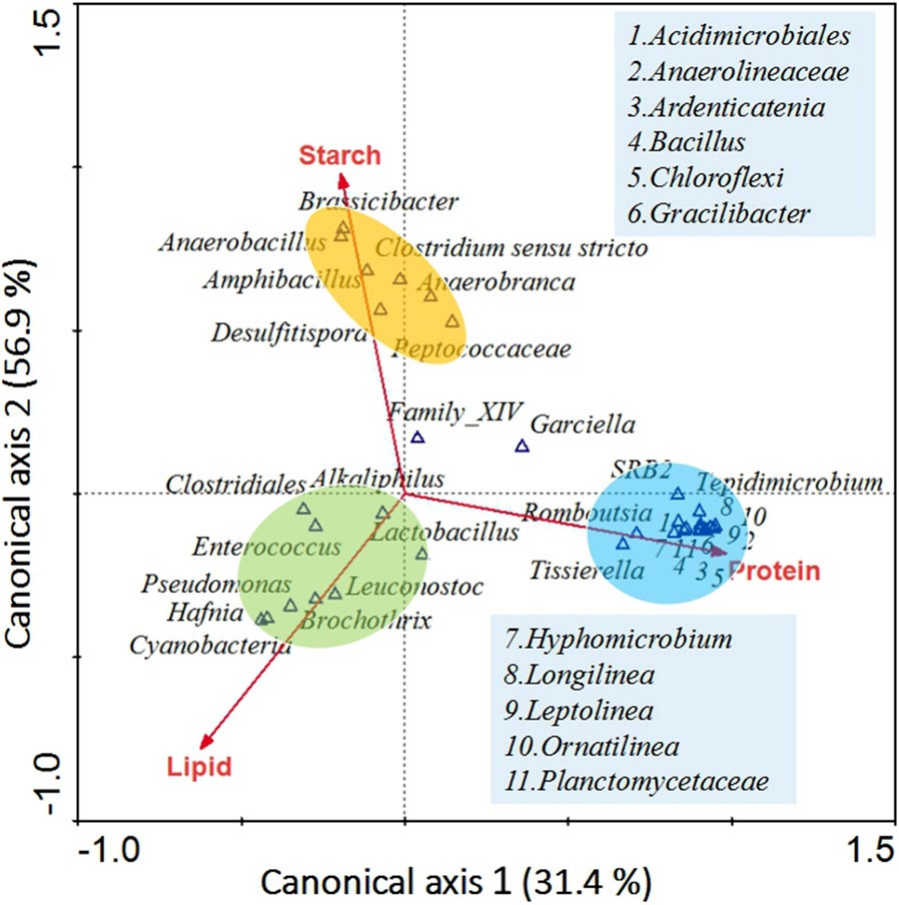
CCA analysis of the microbial communities and the organic composition between the OS, OP, OF, SP3, and SF3 fermentation groups

To explore how the organic matter composition in solid wastes influenced the structure of the bacterial community in detail, the relationship between organic matter composition and typical genera was explained by CCA (Fig. 6). The typical genera of *Anaerobacillus*, *Clostridium*, *Amphibacillus*, and so on, were all located close to the starch, indicating that these genera could be enriched by the feedstock with high starch content (SP3 and OP). With increased lipid content in the feedstock, the dominant genera like *Hafnia*, *Brochothrix*, and *Leuconostoc* were enriched in SF3 and OF samples. In the OS sample, the dominant genera like *Hyphomicrobium*, *Brassicibacter*, *Gracilibacter*, *Ornatilinea*, and *SRB2* were more likely to be enriched, indicating that these genera probably had a close relationship with protein degradation. These results indicated that the structure of the bacterial community was significantly influenced by the organic components in the substrates and thereby generated particular products. For example, the Clostridiales, which was dominant in the starch-enriched substrate, contributed significantly to butyrate production [44], while *Anoxybacillus*, a kind of anaerobic bacteria that only consumes carbohydrate, produced formate, lactate, acetate, and ethanol in low concentrations [45]. *Ornatilinea*, which was abundant in the OS fermentation group, was able to consume a variety of protein substrates to produce valerate as the main product [46]. The intersection angle between starch and canonical axis 1 (Fig. 6) was smaller than the factors of lipids and protein, suggesting that starch was more important than other factors in determining the typical genera in the five groups. The close relationship between protein and the bacterial community mainly explained the enrichment of some specific genera in the OS group.

**Fig. 7:**
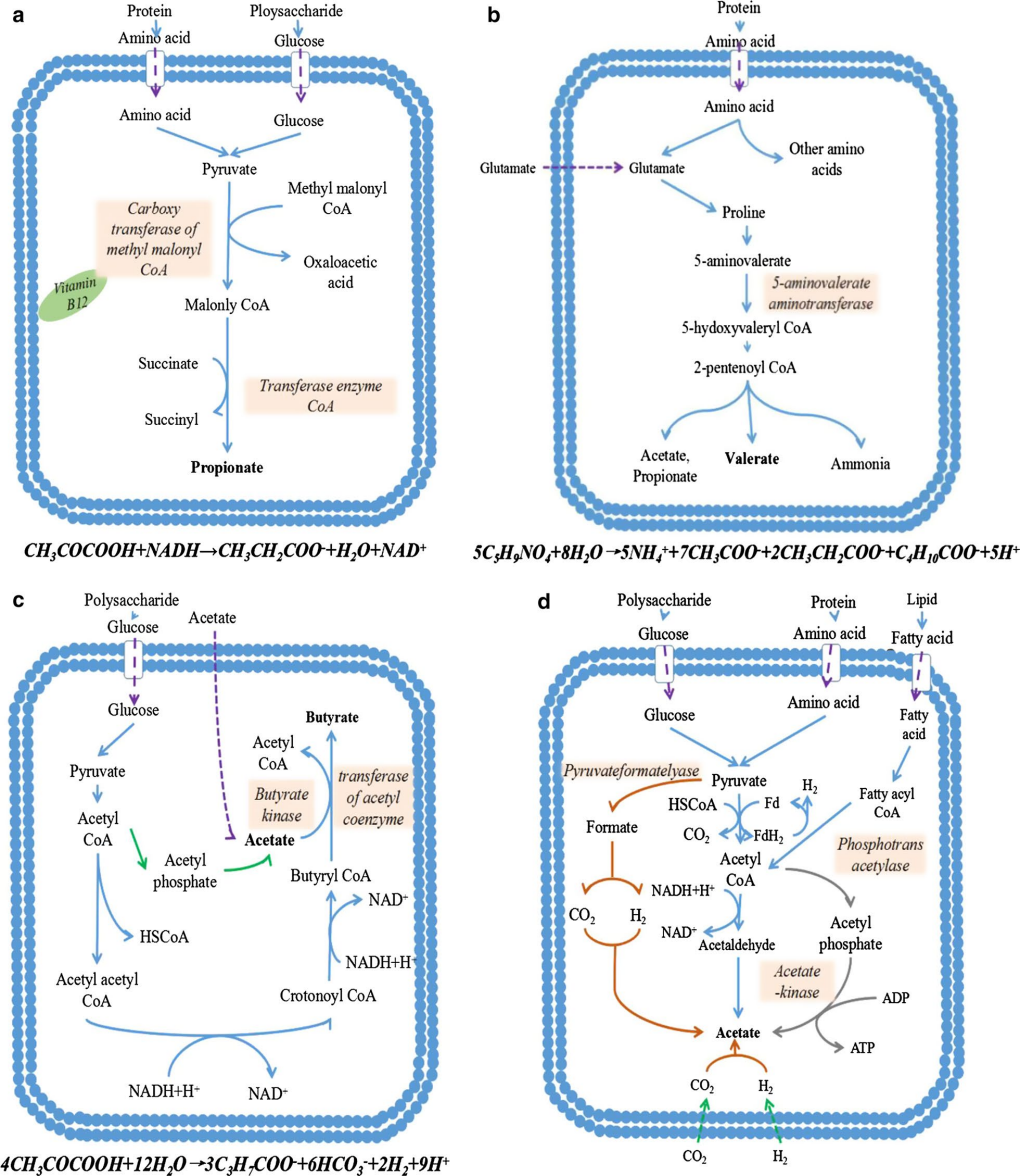
The synthetic pathways of propionate (**a**), valerate (**b**), butyrate (**c**), and acetate (**d**). *Dotted arrows* represent the transport pathway of substrate from extracellular environment to intracellular cytoplasm; *solid arrows* represent the synthetic pathway of single VFA. *Arrows in different colors* represent different synthetic pathways

To further investigate how the different substrates determined the VFAs distribution, the VFAs synthesis pathway was analyzed in detail (Fig. 7). Lipid, starch, and protein are transformed to pyruvate as the common intermediate through a series of biochemical reactions, and then transformed into downstream intermediates. Therefore, pyruvate can be considered as the core and starting point of the VFAs metabolic pathway network with different substrates. The metabolic pathways and the theoretical chemical equations for propionate, butyrate, and valerate are listed in Fig. 7.

Some propionate-producing bacteria, like Planctomycetaceae and *Brassicibacter*, were enriched by consuming a large amount of protein (Fig. 6). From Fig. 7a, propionate was generated through two steps from pyruvate, and vitamin B12 was a co-factor to carboxyl transferase of methyl malonyl CoA, which was involved in the conversion step of pyruvate to malonyl CoA. Vitamin B12 could not exist alone in strong acidic or alkaline conditions unless it combined with protein [47], and the lack of protein would limit the combination of vitamin B12, thus inhibiting propionate generation. The presence of vitamin B12 might benefit propionate accumulation.

Valerate production was related to the conversion of glutamate, which was generated from protein (Fig. 7b). Bacteria like *Bacillus* reduced glutamate to proline [48] and then further reduced it to 5-aminovalerate. Finally, 5-aminovalerate was fermented by bacteria like *Brassicibacter* via 5-hydoxyvaleryl-CoA and 2-pentenoyl-CoA to valerate. Ammonia, acetate, and propionate were also produced simultaneously during fermentation. The pathway in Fig. 7b also demonstrates that the consumption of protein could promote the generation of acetate, propionate, and valerate.

The increase in butyrate that was observed with the addition of starch is possibly related to the type of butyrate-producing bacteria and the acetate concentration (Fig. 7c). It was reported that most butyrate-producing bacteria preferred glucose as their substrates [49], and the enzymes related to butyrate production were mainly transferases of acetyl-coenzyme and butyrate kinase. Over 50% of butyrate-producing bacteria, like *Clostridium* sensu stricto and *Brochothrix*, have both enzymes, which means that they could produce butyrate through the transformation of intracellular acetate [50]. However, some butyrate-producing bacteria could only generate butyrate by using extracellular acetate because of the absence of butyrate kinase. In this study, the SP3 and OP groups had abundant acetate and high concentrations of glucose as extracellular substrates for butyrate-producing bacteria, thus offering a good environment for butyrate generation.

Bacteria related to acetate production were extensively distributed in the fermentation. For example, bacteria like *Ornatilinea* and *Gracilibacter* could produce acetate by consuming the protein or carbohydrate substrates. Moreover, the process of acetate generation and conversion are complex. Figure 7d shows that acetate was generated not only by the conversion of acetyl-coenzyme A, but also through H_2_ and CO_2_ transformation by homoacetogenesis and acetaldehyde transformation by Saccharomycetes in the VFA pathway [51]. The extensive distribution of acetate-producing bacteria and their various metabolic pathways made acetate the major VFA produced during acidogenic fermentation.

## Conclusion

Substrate composition significantly influenced the VFAs production and distribution in acidogenic co-fermentation, as observed by the addition of different types of organic matter. In all cases, acetate was the dominant product in acidogenic fermentation, regardless of the substrate composition. The fermentation profiles and metabolic pathway analysis revealed that the addition of carbon-rich substrates significantly enhanced butyrate and ethanol accumulation, while protein-rich substrates substantially favored propionate and valerate generation.

The abundance of the Bacilli class increased dramatically when potato peel waste was added to the sludge, while the *Clostridia* class rose significantly with the addition of food waste. These trends indicate that the organic matter composition significantly influenced the bacterial community structure, and selectively enriched specific acidogenic bacteria during anaerobic fermentation. The novel findings in this study are very helpful for substrate selection and parameter control for future selective VFAs production by acidogenic co-fermentation from waste activated sludge.

## Additional files

**Additional file 1.** Single VFA accumulation at the end of alkaline fermentation with glucose and bovine serum albumin (BSA).

**Additional file 2.** Content of bacteria at genus level in OS, SP3, SF3, OP, and OF fermentation groups (data given as percentages).

## Abbreviations

BSA: bovine serum albumin
CCA: the canonical correspondence analysis
COD: chemical oxygen demand
C/N: carbon-to-nitrogen ration
FID: flame ionization detector
GC: gas chromatograph
OF: the fermentation substrate was only food waste
OP: the fermentation substrate was only potato peel waste
OS: the fermentation substrate was only pretreated sludge
OTUs: operational taxonomic units
PCA: principal component analysis
SF1: the VS ratio of pretreated sludge and food waste was 3:1
SF2: the VS ratio of pretreated sludge and food waste was 1:1
SF3: the VS ratio of pretreated sludge and food waste was 1:3
SP1: the VS ratio of pretreated sludge and potato peel waste was 3:1
SP2: the VS ratio of pretreated sludge and potato peel waste was 1:1
SP3: the VS ratio of pretreated sludge and potato peel waste was 1:3
TN: total nitrogen
TS: total solid
VFAs: volatile fatty acids
VS: volatile solid
WAS: waste activated sludge
