# Supplementary material for: Novel insight into the relationship between organic substrate composition and volatile fatty acids distribution in acidogenic co-fermentation
Source: Biotechnol Biofuels. 2017 May 26;10:137. doi: 10.1186/s13068-017-0821-1 (PMC5446719; doi:10.1186/s13068-017-0821-1)
Supplement: Supplementary file 1 — Additional file 1. Single VFA accumulation at the end of alkaline fermentation with glucose and bovine serum albumin (BSA). [file 13068_2017_821_MOESM1_ESM.docx]

**
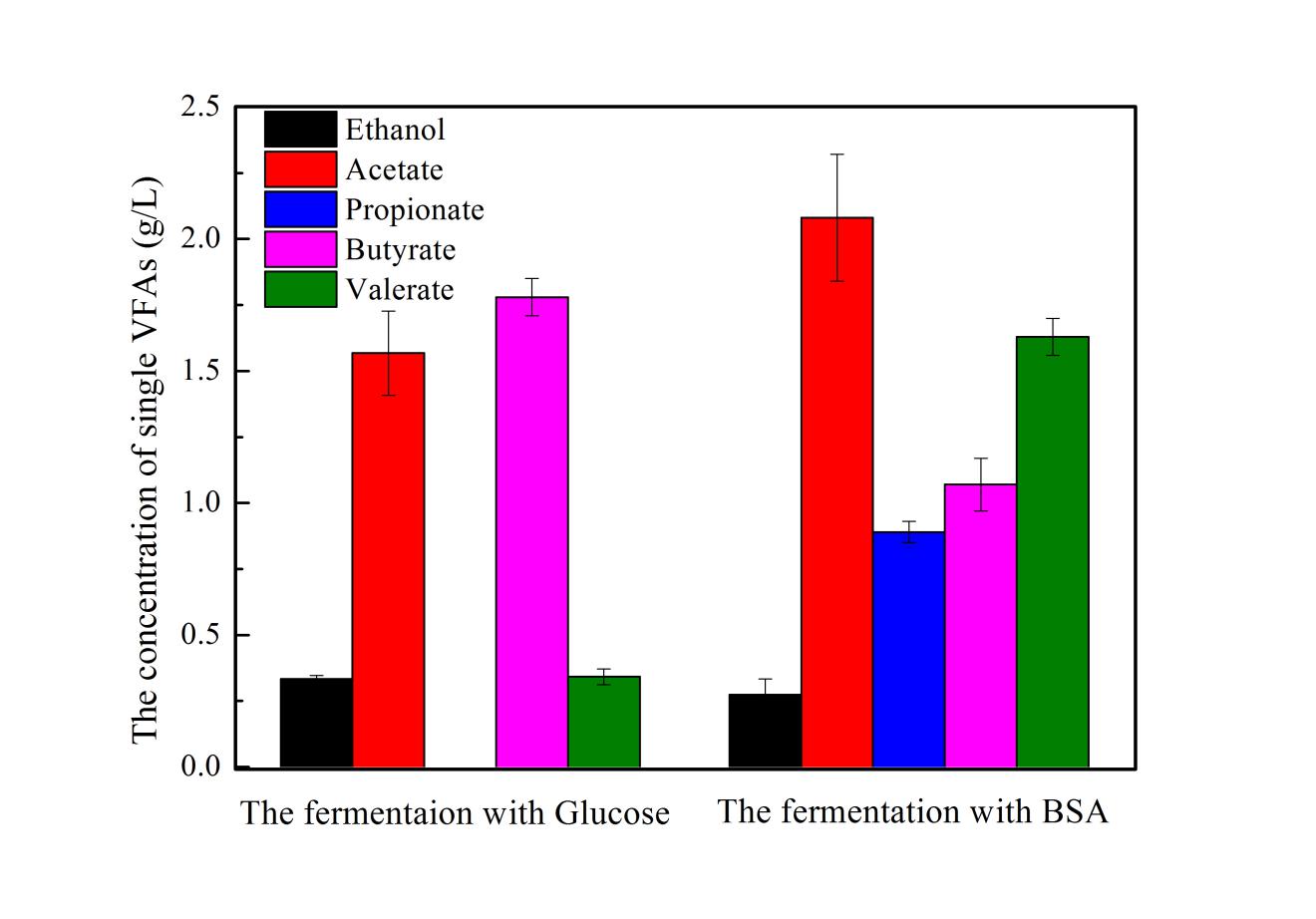
**

Supplementary file 1. Single VFA accumulation at the end of alkaline fermentation with glucose and bovine serum albumin (*BSA*).
