# Supplementary material for: Novel insight into the relationship between organic substrate composition and volatile fatty acids distribution in acidogenic co-fermentation
Source: Biotechnol Biofuels. 2017 May 26;10:137. doi: 10.1186/s13068-017-0821-1 (PMC5446719; doi:10.1186/s13068-017-0821-1)
Supplement: Supplementary file 2 — Additional file 2. Content of bacteria at genus level in OS, SP3, SF3, OP, and OF fermentation groups (data given as percentages). [file 13068_2017_821_MOESM2_ESM.docx]

Supplementary file 2. Content of bacteria at genus level in OS, SP3, SF3, OP, and OF fermentation groups (data given as percentages).

| Taxon (%) | OS | SP3 | SF3 | OP | OF |
| --- | --- | --- | --- | --- | --- |
| Enterococcus | 0.12 | 0.93 | 21.78 | 15.37 | 29.90 |
| Lactobacillus | 8.26 | 7.84 | 40.05 | 0.17 | 0.48 |
| OPB54_norank | 0.09 | 34.16 | 0.01 | 17.87 | 0.01 |
| 9B-04_norank | 0.00 | 27.84 | 0.01 | 0.00 | 0.00 |
| Tissierella | 10.09 | 0.18 | 3.03 | 0.00 | 0.00 |
| Anaerobacillus | 0.00 | 0.64 | 0.082 | 6.22 | 0.10 |
| Brochothrix | 0.00 | 0.02 | 1.77 | 0.10 | 1.94 |
| Leuconostoc | 0.05 | 0.06 | 1.09 | 0.03 | 0.86 |
| Amphibacillus | 0.05 | 0.51 | 0.13 | 1.38 | 0.00 |
| Gracilibacter | 1.27 | 0.01 | 0.04 | 0.00 | 0.00 |
| Clostridium sensu stricto | 1.53 | 5.61 | 3.52 | 14.01 | 41.68 |
| Family XIV | 9.77 | 0.80 | 20.21 | 19.73 | 0.12 |
| Family XI | 1.56 | 0.02 | 0.00 | 0.00 | 0.00 |
| Alkaliphilus | 6.28 | 0.77 | 2.16 | 5.32 | 11.82 |
| Clostridiales | 1.30 | 4.89 | 0.21 | 7.36 | 0.03 |
| Garciella | 5.20 | 2.69 | 1.89 | 1.76 | 0.21 |
| Brassicibacter | 0.04 | 0.51 | 0.03 | 5.63 | 0.00 |
| Peptococcaceae | 1.41 | 1.63 | 0.07 | 2.48 | 0.03 |
| Christensenellaceae R-7 | 1.15 | 0.06 | 0.05 | 0.00 | 0.01 |
| Anaerolineaceae | 5.36 | 0.11 | 0.15 | 0.00 | 0.00 |
| Ornatilinea | 2.40 | 0.02 | 0.03 | 0.00 | 0.00 |
| Ardenticatenia | 1.07 | 0.01 | 0.02 | 0.00 | 0.00 |
| Hyphomicrobium | 1.84 | 0.04 | 0.20 | 0.00 | 0.00 |
| SRB2 | 10.07 | 1.41 | 0.00 | 0.25 | 0.00 |
| Hafnia | 0.00 | 0.00 | 0.09 | 0.00 | 7.57 |
| Synergistaceae | 7.85 | 0.12 | 0.33 | 0.00 | 0.00 |
| Longilinea | 1.24 | 0.01 | 0.02 | 0.00 | 0.00 |
| Armatimonadetes | 1.18 | 0.01 | 0.01 | 0.00 | 0.00 |
| Others | 20.82 | 9.11 | 3.01 | 2.33 | 4.04 |
